# A Circular Material Value Retention Framework for Agricultural By-Product Valorisation

**DOI:** 10.3390/ma19091796

**Published:** 2026-04-28

**Authors:** Roxane Alizad, Yousef Haddad, Konstantinos Salonitis

**Affiliations:** Sustainable Manufacturing Systems Centre, Cranfield University, Cranfield MK43 0AL, UK; roxane.alizad@cranfield.ac.uk (R.A.); yousef.haddad@cranfield.ac.uk (Y.H.)

**Keywords:** circular economy, circular bioeconomy, material value retention, agricultural by-products, crop residue, biomass, bioenergy

## Abstract

While valorisation pathways are increasingly promoted as sustainable solutions, their ability to genuinely minimise environmental harm and contribute to long-term material circularity remains uneven. This study systematically identifies and maps existing valorisation routes across the EU and UK, with particular attention to their environmental performance and economic viability through a material value retention lens. A literature review highlights a spectrum of practices—from soil amendment and composting to bioenergy recovery and bio-based construction materials—each offering different sustainability benefits but varying significantly in their capacity to preserve material quality and function. To address the absence of robust comparative approaches, this paper introduces a novel evaluative framework centred on intrinsic material value retention, a key principle in sustainable and circular material systems. Building on established scholarship, the framework provides a structured means of comparing valorisation options based on how effectively they conserve material properties, particularly in terms of the material’s structural and functional values, and enable high-value reuse. Supported by a dedicated classification tool and a set of guiding questions refined through expert interviews, the framework complements existing environmental assessment methods by foregrounding material circularity. In doing so, it supports more integrated, holistic decision-making for the development of a resilient and sustainable circular bioeconomy. This research is intended for academic audiences and may also be of relevance to industry practitioners.

## 1. Introduction

The agricultural sector generates more than five billion metric tons of agricultural residues annually worldwide [1] from which 305.81 million tonnes were produced in the EU27 and UK in 2020 [2]. Agricultural residues are also commonly referred to as crop residues, biomass, or agricultural by-products—the terminology varies by discipline. This substantial volume of by-products is a burden to farmers in many parts of the world, where they resort to open-field burning as a disposal method. The open-field burning of residues results in significant air pollution, CO_2_ emissions and soil degradation, while also posing health risks to exposed individuals. According to Weldesemayat Sileshi et al. [3] about 458 million tonnes of crop residues are burned each year globally, releasing about 1.24 Mt of CH_4_ and 32 kt of N_2_O in 2019 alone. Importantly, along with the destruction of large quantities of valuable biomaterials, the energy, resources, time, and financial inputs invested throughout the crop’s life cycle, from seed to harvest are also wasted. Consequently, the global scale of agri-waste contributes to significant environmental, economic, and social impacts.

With the emergence of circular practices as part of the circular economy and heightened environmental awareness, the UK banned crop residue burning in 1993 [4] and the EU banned open burning of agri-residues in Regulation N°1306/2013. Despite some remote areas still engaging in illegal burning [5], most crop residues are now disposed of in a more purposeful way. Given its global abundance, valorising agricultural by-products offers a key opportunity to reduce environmental harm while contributing to broader sustainability outcomes.

For this reason, a literature review is carried out to gain a clearer understanding of the valorisation landscape, their environmental and economic implications. With increasing policy awareness, growing consumer interest in circular products and extent of academic engagement, the geographical area of the EU/UK is chosen as the focus for a literature review. Expanding the scope further would introduce too many legal, economic, and environmental variables, complicating meaningful comparison and evaluation. Therefore, the literature review focuses on the EU and the UK, adopting a practical and implementation-oriented perspective.

A review of the literature reveals a lack of comprehensive, cross-sectoral reviews on this topic. Existing reviews either remain confined to disciplinary silos such as bioenergy or soil sciences [6,7], are country-specific [8,9]; focus solely on outcomes like carbon efficiency–carbon emission reduction from processes [10] or emphasise policy while neglecting real-world practices. This gap in comprehensive reviews underscores a limited understanding of which valorisation methods are currently in use and how their sustainability impacts compare.

To address this, the literature review maps the environmental and economic implications of agricultural waste valorisation methods for farmers in the EU and the UK. Building on insights from the literature review, this paper develops and presents a novel value retention framework grounded in Circular Economy principles and the concept of intrinsic material value retention, as put forward by Campbell-Johnston et al. [11]. Building on the contributions of Donner et al. [12] and Cansado et al. [13], the framework offers a more systematic and comprehensive approach to assessing the circularity of agricultural by-product valorisation methods. It enables the evaluation of how effectively different methods retain the structural and functional value of fibrous biomass before its eventual return to the biological cycle.

## 2. Methods

### 2.1. Systematic Literature Review

This study employs the Preferred Reporting Items for Systematic reviews and Meta-Analyses (PRISMA) 2020 method for the needs of the systematic literature review (SLR) to ensure a transparent, comprehensive, and unbiased review of the existing research [14].

**Figure 1:**
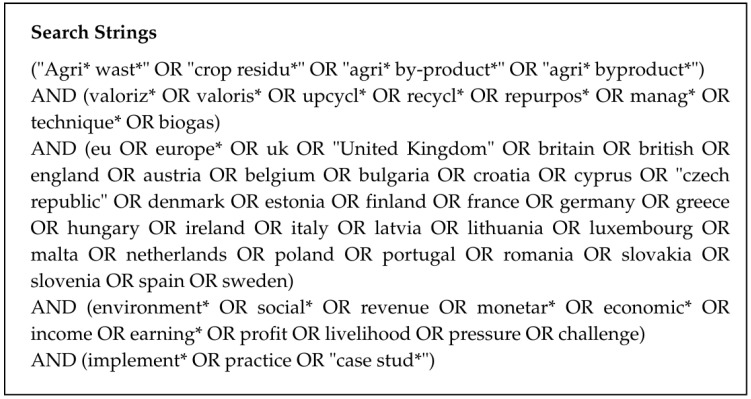
SLR Search Strings.

The databases selected for the search were Scopus, ProQuest, and EBSCO, due to the breadth and relevance of their research coverage. ProQuest and EBSCO were chosen in particular for their accessibility to business-related studies, real-world case studies, and reports. After conducting numerous keyword searches informed by topic-relevant review papers and keyword combinations, the search strings (see Figure 1) were employed for Scopus and ProQuest, yielding the most comprehensive and relevant results. Following several trials, results indicated the need to specify individual countries, as searches limited to ‘EU’ or ‘UK’ yielded significantly fewer studies. The search was limited to English sources, and the publication period was restricted to 2015–2025 to ensure relevance and currency. Pre-2015 studies were excluded after preliminary screening showed limited relevance. Of the 139 articles identified prior to 2015, only 13 were loosely related, with none offering insights beyond those already covered in this paper. Given the evolving nature of agricultural practices and the focus on recent developments, older studies were considered outdated.

**Figure 2:**
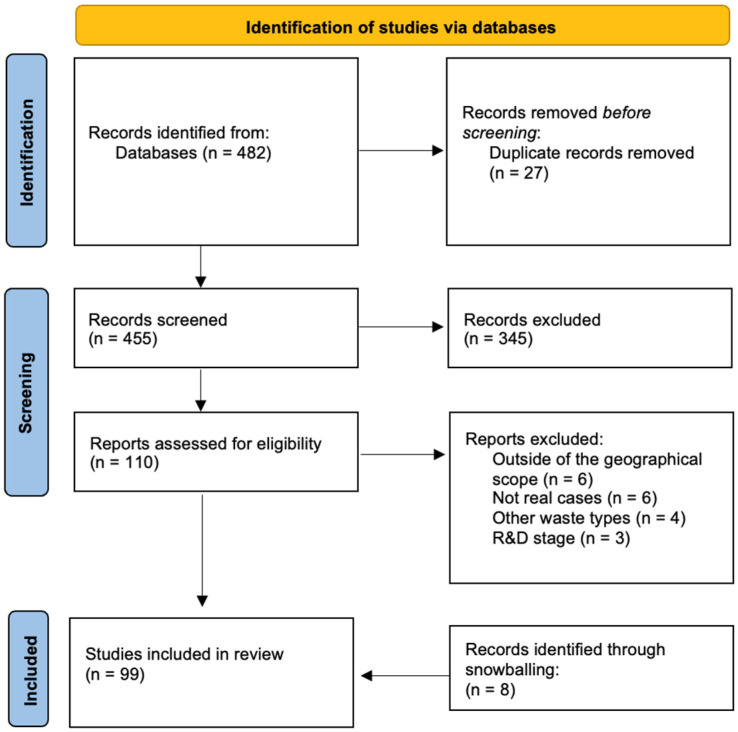
PRISMA Flow Diagram.

The database search produced 231 results from Scopus, 190 from ProQuest, and 61 from EBSCO resulting in a total of 482 documents. The selection process of the included studies is depicted in Figure 2. The criteria used for excluding 345 papers in the eligibility assessment involved reviewing the titles and abstracts to determine broad relevance to the research topic, with conclusions consulted where necessary to clarify inclusion.

### 2.2. Framework Development and Validation

#### 2.2.1. Theoretical Foundations

The core output of this research is the development and refinement of an evaluative framework that ranks agricultural by-product valorisation methods based on their ability to retain intrinsic material value. Grounded in Circular Economy principles and the concept of material value retention proposed by Campbell-Johnston et al. [11], the framework builds on the contributions of Donner et al. [12] and Cansado et al. [13] to offer a more systematic and comprehensive tool for comparing valorisation methods.

The framework was developed through a qualitative, theory-informed process, shaped by both conceptual exploration and empirical input. Following the methodological guidance of Maxwell, the approach proved appropriate given the early stage of development and the limited availability of comparable data across valorisation methods [15]. It allowed for the integration of evidence from the literature alongside expert perspectives, helping to establish a grounded and coherent conceptual foundation.

The framework developed in this study draws on findings from the literature review, which maps the range of valorisation approaches within EU and UK contexts, and is conceptually grounded in established circularity models.

Designed primarily for researchers and practitioners, the framework offers a structured means of comparing valorisation methods through a circularity lens, and towards supporting more integrated and material-focused sustainability assessments.

#### 2.2.2. Subject Matter Expert Validation

To validate the proposed framework, semi-structured interviews were conducted with six subject matter experts selected through purposive and snowball sampling. Participants had expertise in food systems, circularity, agricultural waste valorisation, and sustainability, from both academic and industry backgrounds. The number of interviewees was not predetermined, interviews were concluded when thematic saturation was reached, with no new insights emerging despite the diversity of backgrounds and expertise.

The interviews aimed to assess the framework’s logic, coherence, completeness, and practical relevance, as well as the utility of the accompanying classification tool. Interview questions focused on perceived usefulness, suggested improvements, and identification of missing valorisation pathways. Interview feedback was analysed thematically and integrated into an iterative refinement process, informing the final design of the framework and tool. For detailed interviewee profiles and the full list of guiding questions, see Appendix A.

## 3. Thematic and Geographic Analysis of Research

**Figure 3:**
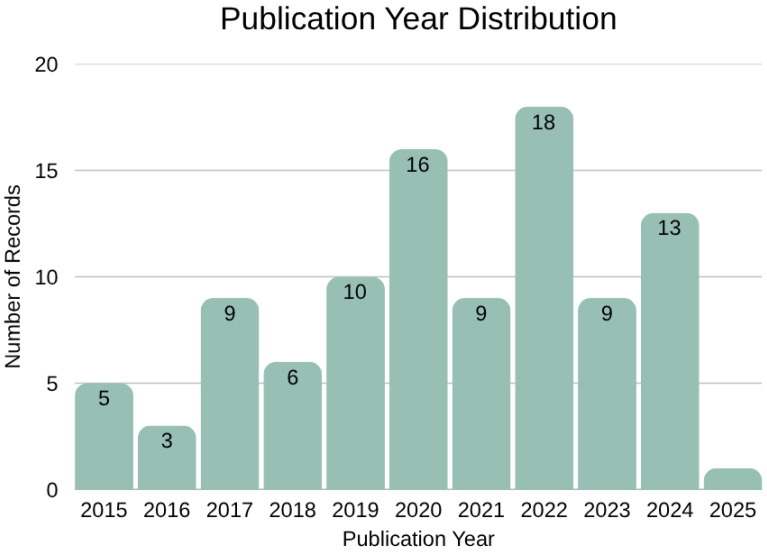
Publication Year Distribution.

This section provides a descriptive and thematic analysis of the literature included in this research paper, highlighting key themes, emerging trends, and geographic representation. A total of 99 records were included, comprising 96 journal articles, two conference papers, and one report. The publication year distribution (Figure 3) does not show a consistent upward trend, contrasting with the overall rise in sustainability and circular economy research, suggesting that agricultural waste valorisation remains a niche area within broader Circular Economy research topics [16]. The data suggest a relatively stable but fragmented body of literature, lacking momentum despite increasing global emphasis on circular systems. The year 2025 is not fully representative of the overall research output, as the data collection was conducted early in the year.

**Figure 4:**
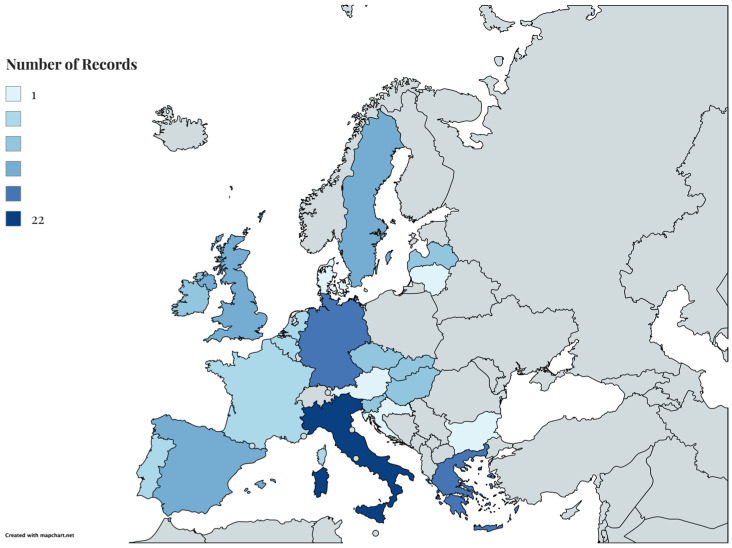
Geographical Distribution.

In terms of geographic distribution (Figure 4), the reviewed studies span a wide range of EU countries, with the highest number of publications focused on Italy, Germany and Greece. This concentration may reflect more active national research agendas and funding structures on sustainable agriculture and waste valorisation. At the same time, this highlights an opportunity for further research in underrepresented EU countries, especially those with significant agricultural activity but limited academic research activity and output in this area.

**Figure 5:**
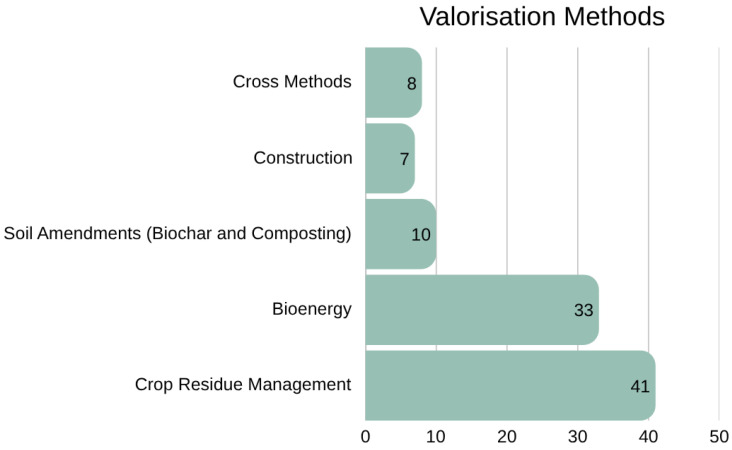
Valorisation Methods.

A thematic analysis was conducted during the screening phase to classify the valorisation methods featured in the literature. The analysis identified four dominant categories: post-harvest crop residue management (41 papers), bioenergy (33 papers), soil amendments; encompassing both biochar and composting (10 papers), construction products (7 papers) and finally, several review papers covered multiple valorisation methods within a single study (Figure 5). While biochar and composting were frequently compared within the same papers, they were grouped together under the umbrella of soil amendments due to their common application in improving soil quality and circular nutrient cycling. Additionally, three papers adopt a more holistic perspective on valorisation methods and their alignment with sustainable or circular approaches. These are discussed in detail in Section 6.

Valorisation methods such as biorefinery and food product upcycling were excluded due to their limited representation in the literature and because identified studies remain solely at the research and development stage [17]. It is also important to acknowledge that the valorisation categories identified in this review are not exhaustive of all real-world practices. Several low-tech or traditional methods such as animal bedding, mulching, mushroom cultivation, and horticultural applications are either underreported in the academic literature or fall outside the scope of academic journals. This reveals a critical disconnect between research outputs and on-the-ground practices, underscoring the need for more applied and field-based research that reflects the full spectrum of existing valorisation activities. Another remark is that studies tend to cluster within specific disciplinary domains, such as energy policy or soil science. While this will be explored further in the following analysis, it suggests a degree of fragmentation in how valorisation methods are studied across fields.

The next section builds on this thematic structure (Figure 5) to assess the environmental and economic sustainability implications of the four dominant valorisation methods: post-harvest crop residue management, bioenergy, soil amendments, and use as construction materials.

## 4. Sustainability Implications of Waste Valorisation

This section maps the environmental and economic implications of the valorisation methods, organised according to the four dominant methods identified through thematic analysis.

### 4.1. Environmental Considerations

**Figure 6:**
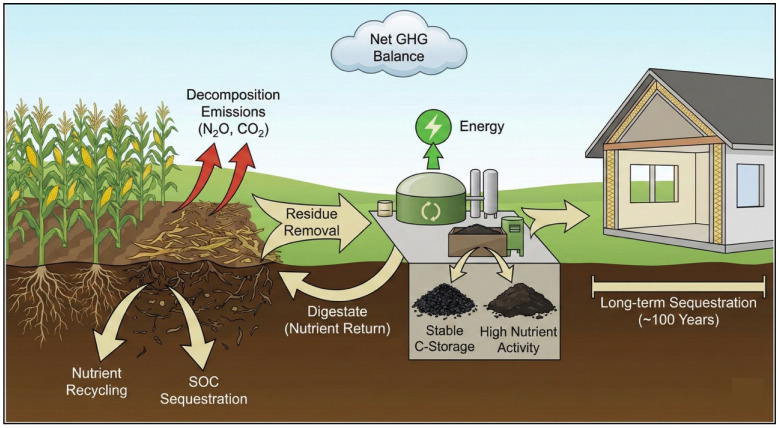
Summary of Identified Environmental Considerations Across Indicative Valorisation Methods.

**Figure 7:**
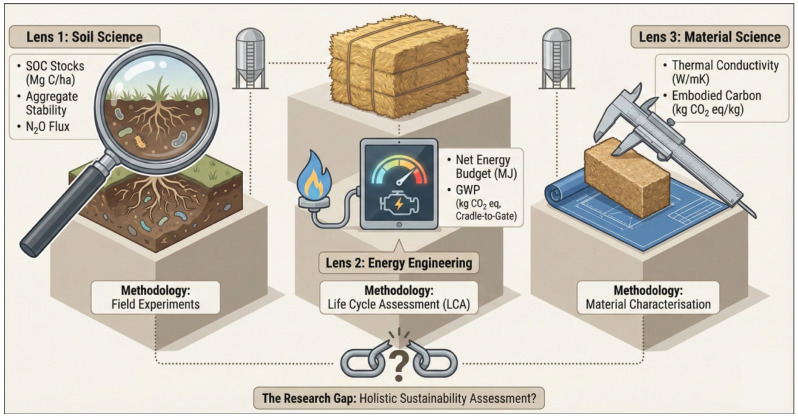
Overview of Methodological Approaches and Identified Research Gaps.

The environmental considerations identified in the literature include climate change impacts (emissions, carbon sequestration, and energy efficiency), land use, resource efficiency, and soil health, with greenhouse gas (GHG) emissions as the primary metric. The main findings are visually summarised in Figure 6, while Figure 7 maps the methodologies used and identifies the corresponding research gaps. In the following sections, the main agri-waste valorisation pathways will be discussed.

#### 4.1.1. Crop Residue Management: Soil Benefits

Crop residue management is widely recognised for its potential to improve soil health through nutrient recycling and organic matter inputs. After harvest, residues are either incorporated into the soil or retained on the surface, where microbial decomposition releases nutrients and contributes to soil organic matter formation [18,19,20,21,22]. This process supports soil fertility and structure [23,24], while improving aggregate stability, infiltration capacity, erosion control, and water-holding capacity [7,18,19]. Additional environmental benefits reported in the literature include weed and pest suppression [20,21] reduced toxic metal availability through straw incorporation [22], and improved soil moisture retention, particularly under warm or arid conditions [21].

Despite these benefits, the sustainability outcomes of crop residue management remain highly variable. Several studies report contradictory findings, reflecting the strong case-specific nature of this practice. Results differ substantially depending on soil type, climatic conditions, residue characteristics, and, most critically, tillage and agricultural management practices. While some studies observe yield improvements over time [25,26], others document initial yield declines and nitrogen losses during the first years of implementation [27,28]. Poeplau et al. [29] further report that residue incorporation does not consistently increase soil organic carbon in warm temperate climates.

Nitrogen dynamics represent a recurring source of uncertainty. Johnson et al. [30] show that residues with high decomposition rates can contribute to nitrogen losses, while Bechini et al. [28] note that slow decomposition often requires supplemental nitrogen inputs to sustain crop productivity, introducing additional costs and management complexity for farmers. Moreover, decomposition processes can generate short-term greenhouse gas emissions, particularly N_2_O, which complicates the overall environmental balance [21].

Overall, most studies position crop residue management as environmentally preferable to open-field burning or landfill disposal [31,32,33]. However, its performance varies widely across contexts [34,35,36], and few studies situate this practice within broader circularity frameworks or assess its interaction with alternative valorisation pathways, such as [37].The inconsistencies observed across the literature underscore the need for systems-based research that captures the complex interactions between soil properties, climate, residue characteristics, and agricultural management practices.

Another key discussion in the literature centres on the carbon sequestration potential of crop residue management [27,38,39,40,41,42]. However, a significant environmental concern is the release of greenhouse gases (GHGs) during residue decomposition. The magnitude of these emissions is influenced by various factors, including soil type, tillage practices, climate, local conditions, and the specific type of crop residue.

Given this sequestration potential, some authors advocate for residue retention as a land-based mitigation strategy for climate change. However, due to the associated emissions and implementation complexities, the viability of this approach is further examined in the following section.

#### 4.1.2. Crop Residue Management: Land-Based Mitigation Strategy

Increased attention to reducing anthropogenic emissions has intensified research into land-based mitigation strategies. One such approach is the retention of crop residues in soil to sequester carbon, positioning agriculture as a contributor to climate targets. This practice, however, competes with other uses of residues within agriculture and the broader bioeconomy, making it a subject of ongoing debate.

Incorporating crop residues into the soil contributes to maintaining Soil Organic Carbon (SOC) stocks by capturing atmospheric carbon and storing it in the soil [43]. It also reduces emissions associated with transporting residues for off-site processing. While Stella et al. [43] argue against residue removal due to its impact on SOC, a wider body of literature explores how residues can be partially removed without undermining sequestration [40]. These studies propose safe removal thresholds informed by soil type, climate, and cropping systems, seeking to strike a balance between soil health and supporting the bioeconomy [44,45,46,47,48,49].

For instance, Hansen et al. estimate that only 26% of Denmark’s straw potential can be removed without jeopardising SOC level [44]. At the EU level, Monforti et al. calculate that 146,000 kt/year of residues could be safely used [45], and Lugato et al. estimate that 15.3 to 50.6 Mt/year of maize stover may be available [46]. Through a life cycle assessment, Monteleone et al. demonstrate that using wheat straw for energy could result in a net environmental gain based on emissions saved and net energy produced [48]. Barrios Latorre et al. further propose that digestate could help offset SOC losses from residue removal [47].

Many of these authors stress the context-specific nature of outcomes, shaped by climate, soil conditions, and agricultural practices [45,46,47,50]. Moreover, Ruijter et al. [51] and Notaris et al. [52] argue that in conventional tillage systems, residue removal shortly after harvest may help prevent GHG emissions from decomposition. Their findings suggest that decomposition-related emissions increase significantly if residues remain on the field beyond ten days post-harvest.

This body of literature reflects a growing interest in finding trade-offs between residue retention and removal, yet it falls short of proposing scalable or unified strategies due to the variability of site-specific findings. Carbon sequestration outcomes are affected by a complex interplay of soil properties, crop types, climate, and practices; many of which remain poorly understood [43,45]. One notable gap is the limited consideration of GHG emissions from retained residues under different tillage systems. While many studies focus on SOC changes, few measure emissions like N_2_O released during decomposition. Under conventional tillage, soil aeration can accelerate decomposition and GHG emissions, undermining sequestration gains [52].

Given that conventional tillage is still widely practiced in the EU, residue retention under such systems may lead to net emissions rather than net carbon gains. Further complicating the picture are uncertainties around carbon saturation levels in soils and the duration of carbon storage. SOC gains tend to plateau over time, with Stella et al. [43] noting a decline in sequestration rates after decades of best practices. By comparison, other applications such as construction materials offer sequestration potential lasting up to 100 years [42,53].

Importantly, uncertainties in baseline data significantly affect modelling outcomes. Dupla et al. find that soil carbon quantification protocols can overestimate carbon stocks in depleted soils by 71% and underestimate richer soils by up to 549%, casting doubt on the reliability of long-term SOC projections [54].

In sum, while crop residue retention has potential as a climate mitigation tool, current evidence is insufficient to support this claim. Further research is needed to assess: (1) long-term impacts of tillage systems and agricultural practices on SOC; (2) soil carbon saturation dynamics; and (3) the accuracy of SOC monitoring protocols (data availability and methods). Advances in these areas are critical to developing effective and scalable land-based mitigation strategies. The following section explores how crop residues are valorised through alternative pathways across agriculture and industry in the EU and UK.

#### 4.1.3. Renewable Energy: Biogas and Others

The environmental implications of converting agricultural by-products into bioenergy, primarily through anaerobic digestion, can be broadly categorised into three areas: emissions, land use, and soil health.

In terms of emissions, biogas systems contribute both to emission reductions and releases. Emission savings result from improved manure and fertiliser management, reduced reliance on agrochemicals, and the diversion of residues from more harmful practices such as open-field burning and landfilling. Diverting manure to biogas production, for instance, significantly curbs methane emissions [6,8,35,55,56,57,58,59]. Digestate, the by-product of anaerobic digestion, substitutes mineral fertilisers and contributes to nutrient recycling [57,60,61]. However, digestate rarely replaces synthetic fertilisers entirely. Most farmers use it in combination with conventional amendments due to concerns over crop yield reduction [62].

Further emission reductions are reported when residues are diverted from conventional crop residue management, particularly open-field burning [56,57,63]. Unlike crop residue literature, which values residue retention for soil health, bioenergy studies often treat residues as an environmental burden. Authors cite emissions from residue decomposition and landfilling as major environmental concerns [57,59]. Digestate is typically promoted as a substitute to mitigate these effects.

Life Cycle Assessments (LCAs) show lower emissions from biogas production compared to open-field burning and fossil fuel use [31,33,56]. Despite its illegality in many EU countries, residue burning persists. Tziolas et al. [33] employ a cradle-to-gate LCA, while Aravani et al. [31] extend the analysis to cradle-to-grave. Both use Global Warming Potential (GWP) as a key metric, underscoring the climate benefits of biogas over conventional fuels [8,56,59,63,64,65,66].

Nonetheless, biogas production emits GHGs during feedstock handling, storage, and transport [58]. Realising net climate benefits depends on balancing emissions with savings, including sequestration by feedstocks. Net GHG savings vary with system design and feedstock type [6]. Poorly managed systems may also lead to nutrient leaching and fugitive methane emissions production [6].

Overall, research on bioenergy tends to be siloed within a single domain, often assuming crop residues are readily available while overlooking alternative valorisation pathways [36,67,68]. Many studies centre on the feasibility and technical performance of bioenergy implementation, with limited engagement in broader sustainability considerations [57,69,70]. This narrow perspective is likely shaped by EU and national climate-neutrality agendas, which position bioenergy as a key decarbonisation strategy. The lack of interdisciplinary input, particularly from sectors involved in alternative residue uses, further limits the quality of sustainability assessments. As a result, environmental considerations often remain narrowly framed and lack the necessary pluri-disciplinarity.

A holistic and comprehensive understanding of potential impacts is essential before implementing policies to avoid unintended consequences. For example, in Germany, policy-driven pressure to scale up bioenergy led to the transformation of small-scale, decentralised plants into large-scale facilities [34]. To sustain these operations, large volumes of feedstock were required, prompting a shift toward intensive cultivation of dedicated energy crops. This unsustainable land use change had adverse environmental consequences and generated competition with food production [68]. These outcomes ultimately forced a policy reversal, with support redirected toward small-scale digesters and a renewed emphasis on using crop residues rather than dedicated energy crops [66].

Regarding soil health, digestate is often promoted as an environmentally beneficial alternative to mineral fertilisers. Field experiments indicate enhanced nitrogen fixation compared to crop residue incorporation. However, nutrient losses can occur if digestate is misapplied, particularly when incorporation timing and technique are not optimised [71].

While biogas from manure and residues generally offers net GHG reductions, the strong policy emphasis on bioenergy has overshadowed alternative valorisation pathways. This narrow framing risks locking in less optimal solutions. Broadening the research lens is essential to ensure more holistic sustainability outcomes and avoid unintended consequences, as demonstrated by Germany’s experience.

#### 4.1.4. Soil Amendments: Biochar and Composting

In addition to biogas digestate, biochar and composting are key valorisation methods for soil amendments. Both enhance soil health and carbon sequestration but differ in their environmental trade-offs.

Both methods increase soil organic matter (SOM), aggregate stability, water retention capacity and erosion control while minimising groundwater contamination and enhancing fertility [72]. However, like crop in residue management, their sequestration potential highly depends on soil type, tillage practices, crop systems, and climate conditions [73,74].

Compost and biochar differ primarily in the types of emissions they release and the specific contributions they make to soil fertility. Biochar has a slow rate of decomposition which gives it a more effective long-term carbon storage potential. In 389 paired field measurements, Han et al. [75] found that biochar significantly enhances soil carbon storage, increasing average SOC levels by 45.8%. However, there is variability in the results and the spatial response of SOC to biochar addition in cropland is still unclear [75]. Borchard et al. [76] also find that biochar addition to the soil decreases soil N_2_O emissions by 28%, however, this is also subject to case specificity. In contrast, composting provides greater nutrient recycling and microbial activity due to its higher availability [77]. However, this comes with a faster rate of decomposition, which entails higher GHG emissions. A cradle-to-farm LCA identifies composting’s primary environmental benefit as nutrient recycling [77].

Biochar and compost thus both offer soil health benefits as well as sequestration potentials. While biochar appears to be a better option for long-term sequestration, compost offers higher nutrient recycling functions for the soil which comes with its associated emissions.

#### 4.1.5. Construction Materials

The environmental impacts of using crop residues in construction depend on the type of residue, product function, and processing stage. Overall, key benefits relate to emission reduction and resource efficiency. Bio-based insulation materials reduce both embodied and operational emissions [42,78] and can store carbon for up to a century [42]. Locally sourcing residues further lowers emissions [78], while materials like biochar can substitute cement and aggregates, offering additional sequestration benefits [79].

These materials also reduce demand for water and virgin resources such as clay, sand, petroleum, and wood [53], helping to mitigate the environmental footprint of the construction sector, which accounts for 40% of global CO_2_ emissions [80]. LCAs show promising results; for example, hempcrete outperforms conventional products across most environmental impact categories [81], and hemp-based insulation panels show a net negative carbon footprint of −4.2 tons CO_2_ per panel [42]. However, full life-cycle assessments, including energy use and recyclability, remain limited. Agricultural by-product valorisation may also help address construction and demolition waste, which makes up 40% of total EU waste [82]. Overall, bio-based construction materials present high potential for reducing emissions and resource depletion, but possible negative trade-offs remain understudied. Further research is needed to fully understand their long-term sustainability performance.

### 4.2. Economic Implications

**Figure 8:**
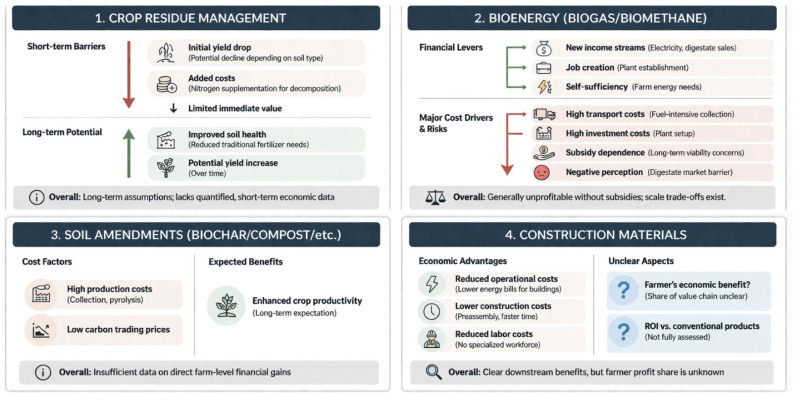
Summary of Identified Economic Implications Across Valorisation Methods.

Economic implications encompass references to the financial impacts of valorisation methods, ranging from indirect effects such as projected yield increases to direct assessments of financial feasibility. These include considerations of subsidies, as well as economic benefits generated for local communities, farmers, and consumers. The main economic findings are visually summarised in Figure 8.

#### 4.2.1. Crop Residue Management

While the reviewed literature does not provide direct or quantified economic assessments, the environmental benefits discussed in the previous section imply indirect economic value for farmers. Improved soil health, for example, could reduce the need for traditional fertilizers, resulting in lower input costs [23,28]. However, due to existing uncertainties in the literature, more studies are needed to support this claim.

While crop productivity [25,26] points to long-term economic returns, Sarkar et al. [21] highlight that the lack of short-term benefits remains a barrier to adoption. Furthermore, Badagliacca et al. [27] find that yields can decline in the initial years depending on soil type, further discouraging implementation. In interviews with Italian farmers, Bechini et al. [28] note that residue incorporation also involves added costs, as farmers often need to supplement nitrogen for effective decomposition.

Overall, the economic impacts of crop residue management remain limited and largely unquantified, with most assumptions pointing toward long-term rather than immediate economic value.

#### 4.2.2. Bioenergy

Bioenergy offers both new income streams and potential cost reductions. Similar to crop residue management, digestate application to soil is expected to increase crop yield over the long term [73]. Establishing new biogas plants also generates employment opportunities [8]. A 2021 statistical report by the European Biogas Association (EBA) estimates a total of over one million jobs worldwide to be created by 2050 when combining both the biogas and biomethane sectors [83]. Additionally, the outputs of biogas production such as digestate or electricity can be sold externally, creating a new revenue stream for farmers [33]. However, in a real-world case study carried out in Greece, Vlachokostas et al. [84] highlight a limited market for digestate, largely due to negative public perception. Self-sufficiency of farmers, particularly in rural areas, is frequently cited as an economic advantage of biogas adoption [6,33]. Nevertheless, the literature does not clearly demonstrate the extent to which biogas plants meet on-farm energy needs or generate surplus energy for external sale.

Profitability in biogas plants does not follow a single standard model, as financial outcomes vary significantly by case [59]. Profitability depends on factors such as feedstock costs, logistical requirements, government subsidies, and overall production expenses. A review on biogas production and sustainable bioenergy recovery by [59]. In a study on biomethane in Germany, Oehmichen et al. [65] find that biomethane from waste and residues can significantly mitigate GHG emissions compared to fossil fuels; however, its production costs often exceed the costs of fossil fuel production. Moreover, bioenergy must compete with other renewable energy sources such as solar and wind [59]. Transportation is consistently identified as the most significant cost factor, primarily due to the fuel-intensive nature of collecting low bulk density biomass from dispersed rural sources. Investment costs also pose notable barriers to adoption [58,63,84]. Importantly, biogas production in both the EU and the UK remains heavily dependent on subsidies [34,35,64], which raises concerns about the sector’s long-term viability once financial support is withdrawn [63]. Although large-scale plants tend to be more profitable, they are not environmentally sustainable due to the reliance on economies-of-scale energy-intensive cropping. In contrast, small-scale farm systems are more sustainable but often economically unviable due to lower yields [34,35,64].

In summary, the literature suggests that bioenergy production in the UK and EU is generally unprofitable and largely dependent on subsidies. Transportation costs represent the most significant financial burden, and while small-scale plants offer environmental advantages, they are typically not economically profitable [6]. At present, the role of biogas in enhancing farm-level revenue remains unclear. Further research involving quantification, standardisation, and direct data collection from farmers is needed better to assess the economic benefits of farm-scale biogas systems.

#### 4.2.3. Soil Amendments

Biochar application is expected to enhance long-term crop yields [73], but economic benefits for farmers remain unclear. High pyrolysis and feedstock collection costs, coupled with low carbon credit prices limit profitability [75]. Profit margins vary by region, soil fertility, and supply chain structure. Though more viable in Europe and North America, the broader economic case for soil amendments lacks robust, quantified evidence.

#### 4.2.4. Construction Materials

The most significant economic benefit of crop residue valorisation into construction materials is reduced operational costs [53,78,81]. These savings stem from decreased energy consumption during building use, owing to the improved thermal insulation properties of bio-based materials [85,86,87]. Lower emissions also translate into reduced utility bills, as less energy is required for heating and cooling.

In a case study, Mutani et al. [78] found that wheat straw-based materials led to lower construction costs and shorter build times compared to conventional options. Products can be preassembled, minimising both labour and financial inputs. Additionally, they do not require a specialised workforce, further lowering labour costs. Government financial support for green building materials is also increasing [78,85,86].

Initial findings suggest that construction materials derived from agricultural by-products can reduce operational, construction, and labour costs. However, profit margins, ROI, and upfront capital requirements remain insufficiently assessed. The role of farmers in this value chain is also unclear, as is the extent to which they benefit economically from this valorisation method.

## 5. Research Gaps

Findings from the literature review indicate that sustainability implications of agricultural waste valorisation are highly context-dependent and vary significantly across methods. While environmental benefits such as emission reductions, soil health improvement, and carbon sequestration are frequently highlighted, these are often accompanied with trade-offs and uncertainties. Economic outcomes remain underexplored and inconclusive, with most benefits assumed rather than measured.

**Figure 9:**
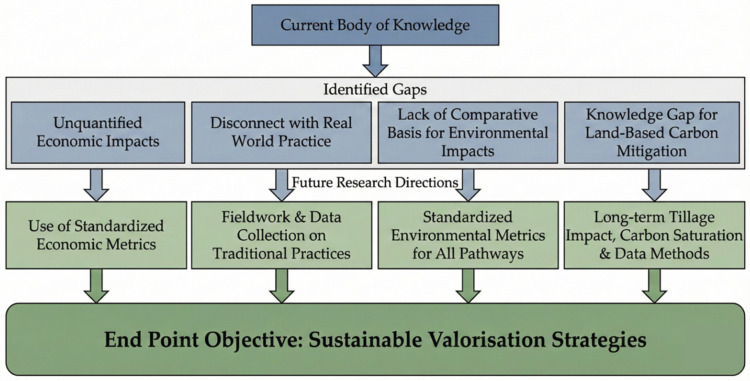
Research Gaps and Future Directions.

Future research should prioritise farmer-centred approaches, adopt standardised metrics to enable comparison across methods such as with life cycle assessments, and bridge the knowledge gap to support land-based carbon mitigation strategies. Applied and field-based research is essential to capture the diversity of on-the-ground valorisation practices, ensuring policy and innovation efforts reflect reality. Figure 9 illustrates the current gaps and emerging directions.

Perhaps most significantly, and central to the development of the framework in the following section, the literature review reveals a pressing need to integrate cross-disciplinary perspectives. A more integrated, evidence-based, and cross-sectoral approach is essential to fully understand the long-term sustainability potential of various valorisation pathways. Currently, comparative analysis is limited by the lack of common evaluative frameworks; existing studies often differ in the data, values, and indicators they use, rendering comparisons between methods difficult, such as between [30,42,71,72] where the indicators and values lack a common evaluative basis.

This gap is also identified in the wider literature. Agnihotri et al. [88] stress the need for standardised frameworks to assess cascading systems in the circular bioeconomy. They argue that the lack of uniformity in current tools hinders decision-making and stakeholder collaboration. Efforts such as those by van Selm et al. [37], who use LCA to compare GHG emissions and agricultural land use impacts of animal feed, compost, and biogas, mark a step in the right direction. Yet these studies also demonstrate the complexity of conducting cross-method comparison, highlighting the multitude of factors and data challenges involved. These findings underscore the need for the research community to develop more holistic tools and frameworks that allow different valorisation methods to be evaluated on a comparable basis. The gap in current approaches is significant, and this paper contributes a first step toward narrowing it by building on established work in the circularity literature and advancing it in a more systematic way.

The next section addresses this need directly by introducing a decision-making framework that evaluates agricultural by-product valorisation pathways through the lens of intrinsic material value retention.

## 6. Framework for Improved Material Value Retention in the Valorisation of Agricultural By-Products

### 6.1. Review of Existing Circular Economy Approaches to Material Value Retention

As part of the literature review, only a limited number of studies were found to adopt a more integrative perspective on comparing valorisation methods.

Nicastro et al. [89] offer important insights into the legal dimensions of valorisation, particularly through their engagement with the Waste Framework Directive. Their work provides a valuable foundation by situating agri-food waste within existing policy hierarchies, encouraging more resource-efficient uses. However, as new technologies and valorisation methods continue to emerge, there is growing recognition that existing regulatory hierarchies do not fully reflect the evolving landscape of the circular bioeconomy.

Donner et al. [12] as part of the EU-NOAW Project, propose a value pyramid that maps broad trajectories in agro-waste valorisation. Their work is a meaningful step toward visualising sectoral trends and future value chain potential. The pyramid captures a spectrum of material uses, from energy to bio-based products. However, some categories, such as “range of biomaterials” remain general, and the framework does not yet offer a comparative ranking of methods within these categories based on circularity or value retention.

Cansado et al. [13] propose a hierarchy for the handling of biomass waste. The value of their contribution lies in the wide coverage of different valorisation pathways, irrespective of geography or technology readiness. While they offer a more structured approach, it similarly includes an undifferentiated category of “industrial uses,” which is not broken down further in terms of material outcomes nor assessed in relation to principles of circular material value retention.

Collectively, these works lay the groundwork for advancing circular thinking in agricultural by-product management. The Waste Framework Directive continues to serve as a baseline reference, while Donner et al. [12] integrate circular economy logic by drawing on concepts from the Ellen McArthur Foundation [90]. Cansado et al. [13] mapping of diverse uses, from polymers and plastics to textiles, paper, packaging, furniture, and activated carbon has directly informed the categorisation of methods in this paper.

However, despite these valuable foundations, the literature still lacks a comparative hierarchy that ranks a wide range of valorisation methods based on a consistent rationale linked to circular material use. The framework developed here addresses this gap by adopting intrinsic material value retention as its organising principle. While not intended as a comprehensive sustainability assessment, this perspective allows for a more structured, material-focused comparison of valorisation methods. It draws conceptual grounding from the work of Campbell-Johnson et al. [11], who offer a general model of cascading flows and value retention in the circular economy. Although their model is not applied directly to the bioeconomy nor agricultural residues, it provides a valuable theoretical foundation that this framework and accompanying classification tool builds upon, adapting and refining it for application in the circular bioeconomy.

Indeed, the criteria underpinning this intrinsic material value retention hierarchy are grounded in two core principles. First, that the valorisation method allows the material to remain in the economy for as long as possible before returning to its biological cycle, in line with the circular economy principles outlined by the Ellen McArthur Foundation [90]. Second, that the material’s structural and functional value is preserved or enhanced throughout the process. Structural value refers to the physical, fibrous or compositional integrity of the material. Its capacity to maintain form, strength or original molecular structure that allows it to be used in new products with minimal transformation. While functional value pertains to the useful properties or bioactive characteristics of the material that can be either extracted or preserved to serve a new function, even if the structure changes. These two criteria serve as the conceptual foundation for the framework proposed in this paper and build directly on the cascading model of value retention articulated by Campbell-Johnson et al. [11], particularly the discussion in Section 4.1. In this sense, the framework developed is theory-based and therefore not geographically constrained, provided that relevant local contextual factors are taken into account.

### 6.2. Proposed Circular Value Retention Framework for Agricultural By-Products

**Figure 10:**
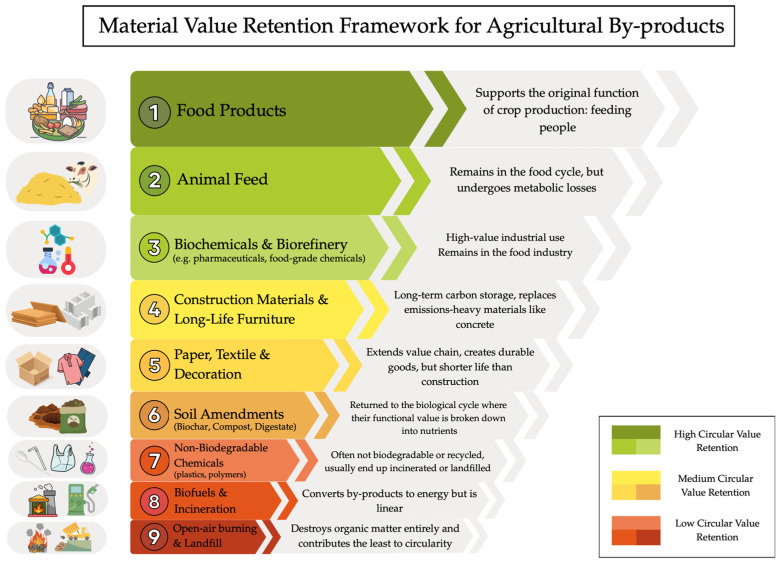
Circular Value Retention Framework.

Following this logic, the valorisation pathways for agricultural by-products are organised in the circular value retention framework presented in Figure 10. This framework consolidates all valorisation routes identified in the literature [13], including applications such as furniture, packaging, textiles, polymers, and activated carbon. Some of these categories were also highlighted by expert interviewees, for example Interviewee 4 explicitly highlighted the need to include activated carbon.

At the top of the hierarchy is the valorisation of by-products into food for human consumption. While absolute waste prevention remains the most desirable outcome, as emphasised by Cansado et al. [13] as well as Interviewees 4 and 5, agricultural systems inevitably generate residual materials. In these cases, the most effective strategy for preventing such residues from becoming waste is to keep them within the human food chain. Interviewee 3 emphasised that this approach aligns directly with the primary purpose of agricultural production, which is to feed people. Donner et al. [12] and Cansado et al. [13] also reflect this prioritisation in their use of the waste hierarchy, where prevention is regarded as the most favourable outcome within both environmental and circular economy frameworks.

The next preferred pathway is the conversion of by-products into animal feed. Although still part of the food system, animal feed ranks slightly lower than human food because of “metabolic losses”. As Interviewee 3 explained, the quantity of product obtained from animals is always less than the quantity of feed originally provided. Interviewee 4 also noted that certain by-products may require cleaning or processing in order to be safely used as feed. Nonetheless, Cansado et al. [13] identifies several by-products that can be safely used for animal nutrition under appropriate conditions. Together, these two top-tier valorisation methods support the circular economy principle of preventing waste while retaining high intrinsic material value.

The following tier includes biorefinery and biochemical applications, particularly those that serve the pharmaceutical or food industries. These applications retain significant functional value and contribute meaningfully to societal needs, although they require substantial processing. For this reason, they are ranked below food and feed, despite being aligned with the prevention category due to the high value they generate.

Next in the hierarchy are pathways that extend the lifespan of materials through durable goods, including construction materials and long-life furniture. Construction applications provide long-term carbon storage and typically remain in use for more than fifty years [42]. Furniture can also function as a mid-tier valorisation route when designed for durability and long-term use.

Below these are applications such as paper, packaging, textiles, and decorative products. These pathways also create value-added products, yet they generally involve more intensive processing and result in goods with shorter lifespans. The additional processing tends to degrade the structural integrity of the original material. Although these goods extend the value chain, they ultimately return to the biological cycle as compost, digestate, or biochar, where their functional value is reduced to nutrients [91].

A subsequent tier includes industrial biochemicals such as bioplastics, polymers, and activated carbon. Although these materials are sometimes described as circular because they are bio-based, empirical evidence suggests otherwise. Most bioplastics today are incinerated or landfilled, and only a small proportion is truly biodegradable under real-world conditions [92,93]. Waste management systems do not yet have the capacity to process these materials separately, and biodegradability varies widely across environmental settings. Activated carbon also undergoes substantial processing, and in most cases cannot be reintegrated safely into the biological cycle unless it is specifically designed to do so, for example through biochar applications. Consequently, unless these materials are demonstrably biodegradable or able to enter a safe and effective recycling loop, they are ranked below soil amendments.

Soil amendments, such as compost, digestate, and biochar, represent a transitional point in the hierarchy. Although they do not preserve the structural form of the material, they still support nutrient cycling by returning organic matter to soils. This biological reintegration makes them a preferable option relative to more linear pathways.

Near the bottom of the hierarchy are biofuels and incineration. These processes convert organic material into energy and therefore serve a functional purpose, yet they destroy the structure of the material. In their hierarchies, Donner et al. [12] and Cansado et al. [13] also classify these valorisation methods below material-preserving applications “range of biomaterials” and “industrial uses”.

Finally, the least desirable outcomes are landfilling and open-air burning, which completely destroy organic matter and offer no contribution to circularity.

### 6.3. Scope and Limitations of the Framework

Two conditions must be met for this framework to function effectively. In retaining material value, the end product’s design plays a crucial role. Products must be made with natural components and be designed for disassembly and safe reintegration into the soil at end-of-life. For example, chemical additives should be replaced with biodegradable binders and pigments in construction and textiles. The importance of product design for end-of-life reintegration has also been underscored in the works of Amato et al. [94] and Vural Gursel et al. [95]. Additionally, the entire value chain must be structured to ensure recovery, collection, and dismantling (e.g., after demolition or garment use) so that materials are truly returned to the soil.

Limitations emerged from both expert interviews and the literature. A key concern was the degradation of material quality through repeated processing, which may compromise structural value and raise environmental costs such as GHG emissions (Interviewee 5). Climatic conditions also affect usability, particularly in humid regions where timely collection is critical to prevent spoilage (Interviewee 4). Declining crop quality, driven by climate change, further reduces the nutrient content and functional value of by-products, limiting their potential for valorisation (Interviewee 5).

Although the Circular Bioeconomy is framed as a pathway to improved land, water, and resource efficiency [96], circularity alone does not guarantee environmental benefit. Interviewees 1, 4, and 5 warned against “textbook” applications of circularity that overlook broader trade-offs, such as increased energy use or toxic by-products. Amato et al. [94] found that innovative circular pathways can result in higher impacts than conventional ones, while Vural Gursel et al. [95] advocate for pairing circularity metrics with comprehensive risk and sustainability assessments.

Promisingly, Rovira-Cal et al. [97] offer a multi-criteria tool combining economic, environmental and social dimensions, though it remains limited to biorefineries. In this context, the material value retention framework presented in this paper is intended to complement, rather than replace, comprehensive sustainability assessments. It provides a focused circularity lens that can be embedded within broader evaluation frameworks. By enabling a more in-depth, systematic, and transparent understanding of how the structural and functional value of fibrous materials is retained, an aspect central to circular thinking, the framework enhances the analytical depth of existing approaches. In this way, it supports more comprehensive and informed decision-making when used alongside methods such as Life Cycle Assessment (LCA). Accordingly, it does not assess economic feasibility, commercial viability, or wider environmental and social impacts, nor does it address effects on farmers, labour conditions, or local communities. Instead, it offers a targeted tool for evaluating intrinsic material value retention, strengthening the circularity dimension of more holistic sustainability assessments.

### 6.4. Accompanying Classification Tool & Guiding Questions

Accompanying the framework, a classification tool and a set of guiding questions are proposed to support its application. As discussed earlier, the framework developed in this paper is not intended to serve as a standalone decision-making tool. Rather, it complements more comprehensive sustainability assessments by offering a material value retention perspective. The classification tool presented here is a first step toward evaluating the extent to which a given valorisation method retains the intrinsic value of agricultural by-products.

**Figure 11:**
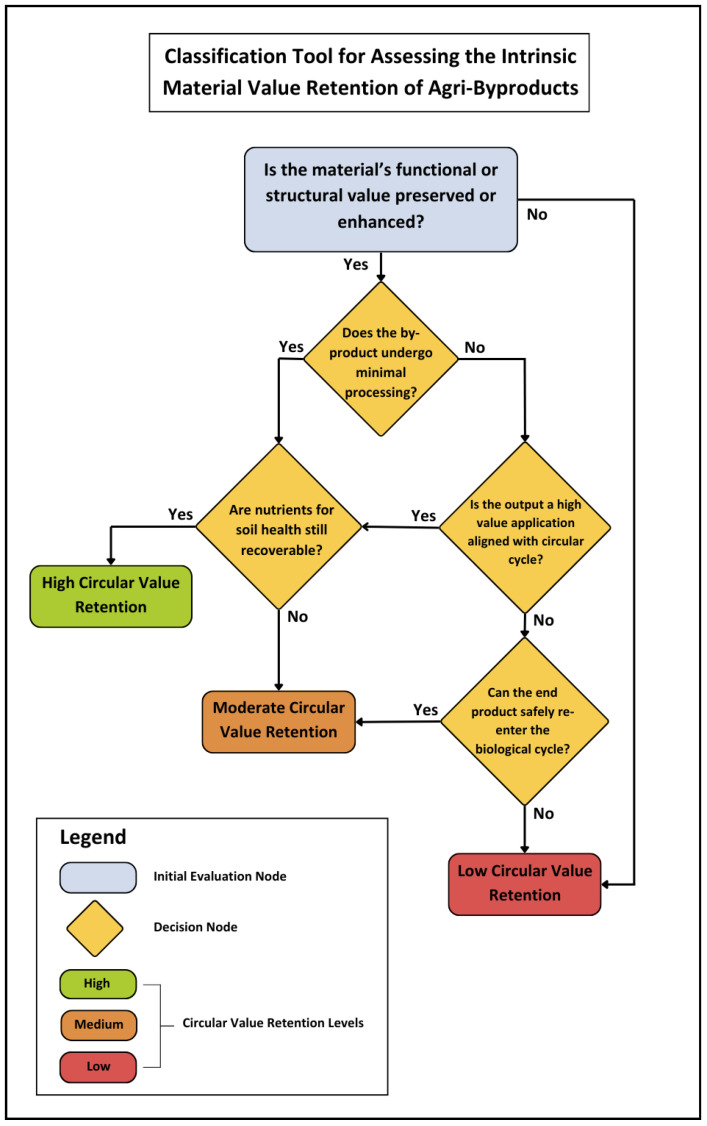
Classification Tool for Evaluating the Intrinsic Material Value Retention.

Given the wide range of potential products and methods, the tool is intentionally broad. It is designed to provide general guidance and prompt reflection on the extent to which functional and structural value is preserved throughout the valorisation process. The classification process presented in Figure 11, begins by asking whether the method preserves or enhances either the functional or structural value of the by-product. It then assesses the level of processing and the nature of the final product to determine whether it supports circularity. A method that produces a high-value output while also allowing nutrients to return to the soil ranks higher in value retention.

This tool, along with the accompanying questions, is intended to help users think more systematically about both the preservation of material value and the product’s eventual reintegration into the biological cycle. Several limitations remain, as noted by Interviewees 1 and 2. In particular, the classification tool does not specify thresholds for what constitutes “minimal processing.” While this level of detail may be difficult to generalise, it could be developed in future versions on a per-method basis. Nonetheless, interviewees agreed that as a general-purpose tool, this framework has practical relevance.

The primary intended users of this tool are academic researchers, who may build upon it to develop more integrated frameworks that combine circularity with broader sustainability considerations. The tool may also be valuable to industry practitioners involved in the production, processing, or disposal of agricultural by-products, as well as designers who develop products using these materials. All Interviewees confirmed its usefulness in guiding more thoughtful decision-making around valorisation, especially in avoiding the common tendency to default to lower-tier options. Interviewee 1 noted its potential value during the design phase to ensure compatibility with circular end-of-life pathways.

Interestingly, Interviewee 5 highlighted its relevance for climate adaptation, especially in contexts where crop yields are increasingly of lower-quality and therefore discarded. The tool could support more proactive thinking around valorisation options under such conditions. Additionally, Interviewee 2, who works in the food and beverage sector, stressed the need for a practical tool or database to help manufacturers better valorise their food waste while navigating regulatory requirements. This lies outside the scope of the current study but is noted as a valuable direction for future research and tool development.

**Figure 12:**
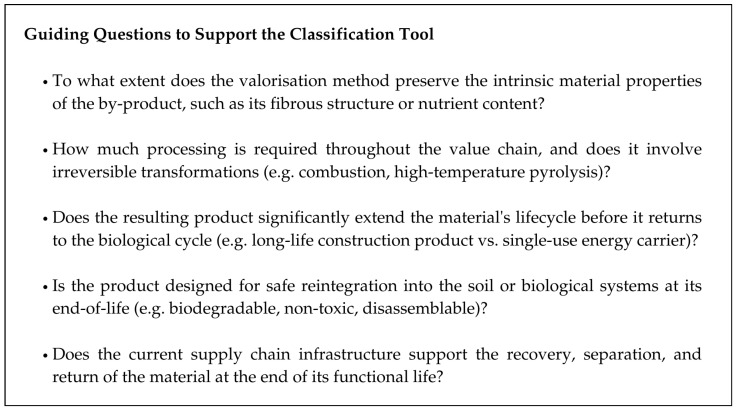
Guiding Questions to Support Value Retention Evaluation.

The questions presented in Figure 12 can guide practitioners and researchers in applying the circular value retention framework introduced in Figure 10. Together, the framework, classification tool, and guiding questions form a qualitative approach to assessing circularity and intrinsic material value in agricultural by-product valorisation.

## 7. Conclusions

This paper identified and assessed four main valorisation methods currently practiced in the EU and UK: (1) post-harvest crop residue management, (2) soil amendments including compost and biochar, (3) bioenergy, and (4) construction materials. These methods differ significantly in their sustainability implications. While crop residue management and soil amendments support soil health and may offer carbon sequestration benefits, they face trade-offs such as nitrogen loss, initial yield declines, and emissions during decomposition. Bioenergy contributes to GHG mitigation and rural energy supply but remains constrained by high production costs, limited digestate markets, and dependency on subsidies. Environmental risks related to energy cropping also undermine its sustainability. Construction materials show promise for circularity, better indoor health performance as well as reduced emissions and operational costs but are underexplored in terms of benefits for farmers.

Despite growing interest in sustainable valorisation, the literature lacks a consistent framework or shared metrics to compare methods meaningfully. To address this, the paper introduces a novel evaluative framework grounded in circular economy principles and informed by the works of Campbell-Johnston et al., Cansado et al., and Donner et al. that ranks valorisation methods based on their intrinsic material value retention [11,12,13].

Building on established scholarship, the framework provides a structured means of comparing valorisation options based on how effectively they conserve material properties, particularly in terms of the materials’ structural and functional values. The accompanying classification tool and guiding questions provide further support for applying this lens.

To the authors’ knowledge, no existing study has systematically evaluated valorisation strategies in this way. While the framework does not assess economic feasibility, production-related environmental impacts, or socio-economic outcomes, it offers a circular materiality perspective intended to complement more comprehensive sustainability assessments. In doing so, it supports more integrated, systemic, and transparent decision-making in the circular bioeconomy.

## Data Availability

No new data were created or analyzed in this study. Data sharing is not applicable to this article.

## Appendix A

### Appendix A.1. Interviewee Profiles and Guiding Questions

#### Appendix A.1.1. Interview Objective

The objective of these interviews was to gather expert feedback on the Intrinsic Material Value Retention Framework and its accompanying diagnostic tool.

#### Appendix A.1.2. Research Questions

The following key research questions were explored through semi-structured interviews:

1. Is the framework logical and coherent?
2. Are you familiar with the EU Waste Framework Directive?
3. How useful are the framework and the decision flowchart in practice?
4. What improvements would you suggest?
5. Are there any valorisation methods that have been overlooked?

#### Appendix A.1.3. Sampling Strategy

Purposive sampling was used to select experts based on their relevance, experience, and domain knowledge. In addition, snowball sampling was employed, with some interviewees recommending additional participants. Five experts were sufficient to reach informational redundancy typical for expert interviews.

#### Appendix A.1.4. Participant Characteristics

A total of five subject matter experts were consulted, representing both academia (*n* = 2) and industry (*n* = 3). All interviewees had demonstrable expertise in circularity, sustainability, food systems, or agricultural by-product valorisation. See table below.

**Table A1:** Interviewee Characteristics.

ID	Sector	Position	Expertise	Sample Sourcing
1	Industry (start-up)	Postdoctoral researcher	Food and agricultural waste valorisation, biotechnology	Purposive
2	Industry	PhD, circularity specialist	Food by-product valorisation	Purposive
3	Academia	Postdoctoral researcher	Circularity, food systems, animal feed valorisation	Purposive
4	Academia	Postdoctoral researcher	Sustainable food systems, agricultural circularity	Purposive
5	Industry	Senior researcher (PhD)	Circular economy in the food sector	Snowball

Note: All interviewees remain anonymous, and institutional affiliations have been removed in accordance with ethical research standards.

## Appendix B

**Table A2:** Table of Included Studies.

N°	Author(s)	Title	Year	Country	Valorisation Method
1	Adi Nugroho et al.	Long-term conservation tillage results in a more balanced soil microbiological activity and higher nutrient supply capacity	2023	Hungary	Crop Residue Retention
2	Allam et al.	Influence of Organic and Mineral Fertilizers on Soil Organic Carbon and Crop Productivity under Different Tillage Systems: A Meta-Analysis	2022	Not determined (ND)	Crop Residue Retention
3	Badagliacca et al.	Early Effects of No-Till Use on Durum Wheat (Triticum durum Desf.): Productivity and Soil Functioning Vary between Two Contrasting Mediterranean Soils	2022	Southern Italy	Crop Residue Retention
4	Bechini et al.	Barriers and drivers towards the incorporation of crop residue in the soil. Analysis of Italian farmers’ opinion with the theory of planned behaviour	2015	ND	Crop Residue Retention
5	Carmona et al.	What do farmers mean when they say they practice conservation agriculture? A comprehensive case study from southern Spain	2015	Spain	Crop Residue Retention
6	Chehade et al.	Evaluation of the Agronomic Performance of Organic Processing Tomato as Affected by Different Cover Crop Residues Management	2019	Pisa	Crop Residue Retention
7	Di Bene et al.	Soil organic carbon dynamics in typical durum wheat-based crop rotations of southern Italy	2016	Southern Italy	Crop Residue Retention
8	Fernandez-Ortega et al.	Double-cropping, tillage and nitrogen fertilization effects on soil CO_2_ and CH_4_ emissions	2024	Spain	Crop Residue Retention
9	Francaviglia et al.	Diversification and management practices in selected European regions. A data analysis of arable crops production	2020	Belgium, France, Germany, Finland, Netherlands, Latvia, Norway and Sweden	Crop Residue Retention
10	García-Palacios et al.	Crop traits drive soil carbon sequestration under organic farming	2018	Sweden, Netherlands, France, Switzerland	Crop Residue Retention
11	Golia et al.	The impact of heavy metal contamination on soil quality and plant nutrition. Sustainable management of moderate contaminated agricultural and urban soils, using low cost materials and promoting circular economy	2023	Northern Greece	Crop Residue Retention
12	Kemda et al.	Environmental impact assessment of hemp cultivation and its seed-based food products	2024	Siena and Tuscany, Central Italy	Crop Residue Retention
13	Korczyk-Szabó et al.	Influence of Crop Residue Management on Maize Production Potential	2024	Slovakia	Crop Residue Retention
14	Lognoul et al.	Impact of tillage on greenhouse gas emissions by an agricultural crop and dynamics of N_2_O fluxes: Insights from automated closed chamber measurements	2017	Belgium	Crop Residue Retention
15	Neto et al.	Effect of Different Irrigated Crop Successions on Soil Carbon and Nitrogen–Phosphorus–Potassium Budget Under Mediterranean Conditions	2024	Portugal	Crop Residue Retention
16	Panagea et al.	Soil water retention as affected by management induced changes of soil organic carbon: Analysis of long-term experiments in europe	2021	Italy, UK, Czech Republic, Hungary, Belgium	Crop Residue Retention
17	Panagea et al.	Impact of agricultural management on soil aggregates and associated organic carbon fractions: analysis of long-term experiments in Europe	2022	Italy, UK, Czech Republic, Hungary, Belgium	Crop Residue Retention
18	Pittarello et al.	Changes in Soil Quality through Conservation Agriculture in North-Eastern Italy	2022	North-Eastern Italy	Crop Residue Retention
19	Poeplau et al.	Qualitative and quantitative response of soil organic carbon to 40 years of crop residue incorporation under contrasting nitrogen fertilisation regimes	2017	Padua, Italy	Crop Residue Retention
20	Raberg et al.	Nitrogen balance in a stockless organic cropping system with different strategies for internal N cycling via residual biomass	2018	Sweden	Crop Residue Retention
21	Simansky et al.	Soil structure after 18 years of long-term different tillage systems and fertilisation in haplic luvisol	2018	Slovakia	Crop Residue Retention
22	Tedone et al.	Effects of Different Types of Soil Management on Organic Carbon and Nitrogen Contents and the Stability Index of a Durum Wheat–Faba Bean Rotation under a Mediterranean Climate	2023	Southern Italy	Crop Residue Retention
23	Valkama et al.	Can conservation agriculture increase soil carbon sequestration? A modelling approach	2020	Southern Finland & Southern Kazakhstan	Crop Residue Retention
24	Wang et al.	Modeling soil organic carbon dynamics and their driving factors in the main global cereal cropping systems	2017	Western Europe	Crop Residue Retention
25	Pergola et al.	Environmental and Energy Analysis of Two Orchard Systems: A Case Study in Mediterranean Environment	2022	Southern Italy	Crop Residue Retention
26	Preiti et al.	Long-term effects of different arable cropping systems on surface erosion processes and C-factor in hilly Mediterranean environment	2022	Calabria, Southern Italy	Crop Residue Retention
27	Stella et al.	Estimating the contribution of crop residues to soil organic carbon conservation	2019	Germany	Crop Residue Retention
28	Searle et al.	Waste and residue availability for advanced biofuel production in EU Member States	2016	EU	Crop Residue Retention
29	Bechini et al.	Drivers and barriers to adopt best management practices. Survey among Italian dairy farmers	2020	Italy	Crop Residue Retention
30	Hansen et al.	Agricultural residues bioenergy potential that sustain soil carbon depends on energy conversion pathways	2020	Denmark	Crop Residue Retention
31	Jensen et al.	Spring barley grown for decades with straw incorporation and cover crops: Effects on crop yields and N uptake	2021	Denmark	Crop Residue Retention
32	Latorre et al.	Managing Soil Carbon Sequestration: Assessing the Effects of Intermediate Crops, Crop Residue Removal, and Digestate Application on Swedish Arable Land	2024	Sweden	Crop Residue Retention
33	Fohrafellner et al.	Quality assessment of meta-analyses on soil organic carbon	2023	ND	Crop Residue Retention
34	Sarkar et al.	Management of crop residues for improving input use efficiency and agricultural sustainability	2020	ND	Crop Residue Retention
35	Hussain et al.	Carbon sequestration to avoid soil degradation: A review on the role of conservation tillage	2021	ND	Crop Residue Management
36	Lugato and Jones	Modelling Soil Organic Carbon Changes Under Different Maize Cropping Scenarios for Cellulosic Ethanol in Europe	2015	ND	Crop Residue Retention
37	Monforti et al.	A new baseline of organic carbon stock in European agricultural soils using a modelling approach	2020	ND	Crop Residue Retention
38	Stella et al.	Estimating the contribution of crop residues to soil organic carbon conservation	2019	Germany	Crop Residue Retention
39	De Notaris et al.	Potential for the adoption of measures to reduce N_2_O emissions from crop residues in Denmark	2022	Denmark	Crop Residue Retention
40	De Ruijter and Huijsmans	A methodology for estimating the ammonia emission from crop residues at a national scale	2019	Netherlands	Crop Residue Retention
41	Monteleone et al.	Cereal straw management: A trade-off between energy and agronomic fate	2015	Foggia, Southern Italy	Crop Residue Retention
42	Ahlberg-Eliasson et al.	Production efficiency of Swedish farm-scale biogas plants	2017	Sweden	Bioenergy
43	Aravani et al.	Agricultural and livestock sector’s residues in Greece & China: Comparative qualitative and quantitative characterization for assessing their potential for biogas production	2022	Greece	Bioenergy
44	Aravani et al.	LCA analysis on the management of typical lignocellulosic agricultural wastes: Case studies and comparison in Greece and China	2024	Greece & China	Bioenergy
45	Auer et al.	Agricultural anaerobic digestion power plants in Ireland and Germany: policy and practice	2017	Ireland & Germany	Bioenergy
46	Blumenstein et al.	Integrated bioenergy and food production-a german survey on structure and developments of anaerobic digestion in organic farming systems	2015	Germany	Bioenergy
47	Bumbiere et al.	What Will Be the Future of Biogas Sector?	2021	Latvia	Bioenergy
48	Busse et al.	Acceptability of innovative biomass heating plants in a German case study—A contribution to cultural landscape management and local energy supply	2019	Germany	Bioenergy
49	Cucchiella et al.	Biomethane: A renewable resource as vehicle fuel	2017	ND	Bioenergy
50	Feiz et al.	Systems analysis of digestate primary processing techniques	2022	Sweden & Belgium	Bioenergy
51	Pari et al.	Fiusis, a biomass power plant fueled exclusively by olive tree prunings. A case study in the Agroinlog H2020 Project	2020	Italy & Greece	Bioenergy
52	Katinas et al.	Analysis of biodegradable waste use for energy generation in Lithuania	2019	Lithuania	Bioenergy
53	Levstek and Rozman	A Model for Finding a Suitable Location for a Micro Biogas Plant Using Gis Tools	2022	Slovenia	Bioenergy
54	Mohrmann and Otter	Categorisation of Biogas Plant Operators in Germany with Regards to Their Intention to Use Straw Pellets as Innovative and Sustainable Substrate Alternative	2022	Germany	Bioenergy
55	Moustakas et al.	Anaerobic digestion for energy production from agricultural biomass waste in Greece: Capacity assessment for the region of Thessaly	2020	Greece, Thessaly	Bioenergy
56	Moustakas et al.	Evaluation of the biogas potential of agricultural biomass waste for energy applications in Greece: A case study of the western Greece region	2021	Western Greece	Bioenergy
57	Egieya et al.	Synthesis of biogas supply networks using various biomass and manure types	2019	Slovenia	Bioenergy
58	Niang et al.	How do local actors coordinate to implement a successful biogas project?	2022	France	Bioenergy
59	Oehmichen et al.	Biomethane from manure, agricultural residues and biowaste—ghg mitigation potential from residue-based biomethane in the European transport sector	2021	Germany	Bioenergy
60	Petig et al.	Linking a farm model and a location optimization model for evaluating energetic and material straw valorization pathways—A case study in Baden-Wuerttemberg	2018	Germany	Bioenergy
61	Renda et al.	Economic Feasibility Study of a Small-scale Biogas Plant Using a Two-stage Process and a Fixed Bio-film Reactor for a Cost-efficient Production	2016	Italy	Bioenergy
62	Steindl et al.	A comprehensive study on the consequences of substituting energy crops by alternative substrates for biogas production in Germany	2023	Germany	Bioenergy
63	Tamburini et al.	Biogas from agri-food and agricultural waste can appreciate agro-ecosystem services: The case study of Emilia Romagna region	2020	Italy (Emilia Romagna)	Bioenergy
64	Tratzi et al.	Liquefied biomethane for heavy-duty transport in Italy: A well-to-wheels approach	2022	Italy	Bioenergy
65	Petravic et al.	Current state of biogas production in Croatia	2020	Croatia	Bioenergy
66	Makepa et al.	Barriers to commercial deployment of biorefineries: A multi-faceted review of obstacles across the innovation chain	2024	ND	Bioenergy
67	Bumharter et al.	New opportunities for the European Biogas industry: A review on current installation development, production potentials and yield improvements for manure and agricultural waste mixtures	2023	ND	Bioenergy
68	Tziolas and Bournaris	Economic and Environmental Assessment of Agro-Energy Districts in Northern Greece: a Life Cycle Assessment Approach	2019	Northern Greece	Bioenergy
69	Venanzi et al.	Use of agricultural by-products in the development of an agro-energy chain: A case study from the Umbria region	2018	Italy	Bioenergy
70	Vlachokostas et al.	Decision support system to implement units of alternative biowaste treatment for producing bioenergy and boosting local bioeconomy	2020	Greece Serres	Bioenergy
71	Marty et al.	Transformation of socioeconomic metabolism due to development of the bioeconomy: the case of northern Aube (France)	2022	Aube, France	Bioenergy
72	Arnau et al.	European Biogas Association (EBA) Annual Report	2022	European Union	Bioenergy
73	Alengebawy et al.	Anaerobic digestion of agricultural waste for biogas production and sustainable bioenergy recovery: a review	2024	Europe & China	Bioenergy
74	Sturmer et al.	Greening the gas grid-evaluation of the biomethane injection potential from agricultural residues in Austria	2020	Austria	Bioenergy
75	Bartzas et al.	Life cycle analysis of pistachio production in Greece	2017	Greece	Soil Amendments
76	Duque-Acevedo et al.	Management of Agricultural Waste Biomass: A case study of Fruit and Vegetable Producer Organizations in southeast Spain	2022	Spain	Soil Amendments
77	Brás et al.	Valorisation of Forest and Agriculture Residual Biomass—The Application of Life Cycle Assessment to Analyse Composting, Mulching, and Energetic Valorisation Strategies	2024	Portugal	Soil Amendments
78	Han et al.	Global soil organic carbon changes and economic revenues with biochar application	2022	ND	Soil Amendments
79	Komnitsas and Doula	Framework to improve sustainability of agriculture in small islands: The case of Pistacia vera L. cultivation in Aegina, Greece	2017	Greece	Soil Amendments
80	Pergola et al.	Sustainability assessment of the green compost production chain from agricultural waste: A case study in southern Italy	2020	Southern Italy	Soil Amendments
81	Dimitrova and Pavlova	Development trends and innovative approaches in agricultural waste management in Bulgaria and the European Union	2024	Bulgaria	Soil Amendments
82	Bogale et al.	Symbiotic and Asymmetric Causality of the Soil Tillage System and Biochar Application on Soil Carbon Sequestration and Crop Production	2023	ND	Soil Amendments
83	Borchard et al.	Biochar, soil and land-use interactions that reduce nitrate leaching and N_2_O emissions: A meta-analysis	2019	ND	Soil Amendments
84	Duque-Acevedo et al.	Sustainability and circularity in fruit and vegetable production. Perceptions and practices of reduction and valorization of agricultural waste biomass in south-eastern Spain	2022	Spain	Soil Amendments
85	Dotelli et al.	Hempcrete Buildings: Environmental Sustainability and Durability of Two Case-Studies in North and South Italy	2020	Italy	Construction Materials
86	Duque Acevedo et al.	Management of agricultural waste biomass as raw material for the construction sector: an analysis of sustainable and circular alternatives	2022	ND	Construction Materials
87	Mutani et al.	Straw buildings: A good compromise between environmental sustainability and energy-economic savings	2020	Italy	Construction Materials
88	Daly and Barril	Biobased Construction from Agricultural Crops: Paper 1—A State of Play of Commercial Solutions in Europe	2024	UK & EU	Construction Materials
89	Daly and Barril	Biobased Construction from Agricultural Crops: Paper 2—Supply Chain Dynamics of European Case Studies	2024	UK & EU	Construction Materials
90	Dams et al.	Upscaling bio-based construction: challenges and opportunities	2023	UK & EU	Construction Materials
91	Dams et al.	Upscaling non-residential bio-based circular construction in the United Kingdom	2021	UK	Construction Materials
92	Pari Luigi	Best available technologies for pruning harvesting	2018	ND	Cross Methods
93	Duque-Acevedo et al.	Sustainable and circular agro-environmental practices: A review of the management of agricultural waste biomass in Spain and the Czech Republic	2023	Spain & Czech Republic	Cross Methods
94	Macura et al.	Effectiveness of ecotechnologies in agriculture for the recovery and reuse of carbon and nutrients in the Baltic and boreo-temperate regions: A systematic map	2019	Europe & North America	Cross Methods
95	Nicastro et al.	Legal Barriers in Sustainable Agriculture: Valorization of Agri-Food Waste and Pesticide Use Reduction	2024	ND	Cross Methods
96	Maina et al.	A roadmap towards a circular and sustainable bioeconomy through waste valorization	2017	ND	Cross Methods
97	Donner et al.	A new circular business model typology for creating value from agro-waste	2020	ND	Cross Methods
98	van Selm et al.	How to use residual biomass streams in circular food systems to minimise land use or GHG emissions	2025	Netherlands	Cross Methods
99	Amato et al.	Environmental sustainability analysis of case studies of agriculture residue exploitation	2021	Central Italy	Cross Methods
